# United States clinical practice experience with eculizumab in myasthenia gravis: symptoms, function, and immunosuppressant therapy use

**DOI:** 10.1007/s00415-024-12569-w

**Published:** 2024-07-25

**Authors:** Ali A. Habib, Andrew J. Klink, Srikanth Muppidi, Anju Parthan, S. Chloe Sader, Alexandrina Balanean, Ajeet Gajra, Richard J. Nowak, James F. Howard, the ELEVATE Study Group

**Affiliations:** 1 University of California, Irvine, CA USA; 2 Cardinal Health, Dublin, OH USA; 3 Stanford Neuroscience Health Center, Palo Alto, CA USA; 4 Alexion, AstraZeneca Rare Disease, Boston, MA USA; 5 Yale University School of Medicine, New Haven, CT USA; 6 Department of Neurology, The University of North Carolina, Chapel Hill, NC USA; 7 Present Address: FibroGen Inc., San Francisco, CA USA; 8 Present Address: Hematology-Oncology Associates of CNY, East Syracuse, NY USA

**Keywords:** Eculizumab, Myasthenia gravis, Chart review, Corticosteroid, Activities of daily living, Clinical practice, Complement inhibition, C5, Immunosuppression

## Abstract

**Background/objectives:**

The phase 3 REGAIN study and its open-label extension demonstrated the efficacy of the complement C5 inhibitor eculizumab in patients with treatment-refractory, acetylcholine receptor antibody–positive generalized myasthenia gravis (gMG). The aim of the ELEVATE study was to assess the effectiveness of eculizumab in clinical practice in adults with MG in the United States.

**Methods:**

A retrospective chart review was conducted in adults with MG who initiated eculizumab treatment between October 23, 2017 and December 31, 2019. Outcomes assessed before and during eculizumab treatment using a pre- versus post-treatment study design included Myasthenia Gravis–Activities of Daily Living (MG-ADL) total scores; minimal symptom expression (MSE); physician impression of clinical change; minimal manifestation status (MMS); and concomitant medication use.

**Results:**

In total, 119 patients were included in the study. A significant reduction was observed in mean MG-ADL total score, from 8.0 before eculizumab initiation to 5.4 at 3 months and to 4.7 at 24 months after eculizumab initiation (both *p* < 0.001). At 24 months after eculizumab initiation, MSE was achieved by 19% of patients. MMS or better was achieved by 30% of patients at 24 months. Additionally, 64% of patients receiving prednisone at eculizumab initiation had their prednisone dosage reduced during eculizumab treatment and 13% discontinued prednisone; 32% were able to discontinue nonsteroidal immunosuppressant therapy.

**Discussion:**

Eculizumab treatment was associated with sustained improvements in MG-ADL total scores through 24 months in adults with MG. Prednisone dosage was reduced in approximately two-thirds of patients, suggesting a steroid-sparing effect for eculizumab.

**Supplementary Information:**

The online version contains supplementary material available at 10.1007/s00415-024-12569-w.

## Introduction

Generalized myasthenia gravis (gMG) is a rare, chronic autoimmune disease characterized by debilitating muscle weakness and fatigue. Common symptoms experienced by patients with gMG may include ptosis, diplopia, dysarthria, dysphagia, dyspnea, head drop, limb weakness, exertional muscle fatigue, and aching muscles [1–4], all of which negatively affect a patient’s ability to live a normal life.

The current international consensus guidance of MG experts includes use of the acetylcholinesterase inhibitor pyridostigmine as first-line treatment for most patients with gMG [5]. For patients with an inadequate response to pyridostigmine, corticosteroids or nonsteroidal immunosuppressant therapies (NSISTs) are recommended (including azathioprine, ciclosporin, mycophenolate mofetil, methotrexate, and tacrolimus) [5, 6]. However, chronic use of corticosteroids is associated with a range of adverse effects, including development or worsening of conditions such as diabetes, hypertension, and osteoporosis [7, 8]. An important treatment goal in gMG is to minimize corticosteroid use while maintaining symptom control [7, 9]; therefore, once patients achieve optimal clinical status, the daily dosage of corticosteroids should be tapered to the minimal effective dosage [6, 7, 9–11]. The consensus guidance also recommends that the treatment goal for patients with gMG should be achievement of minimal manifestation status (MMS; defined as having no symptoms or functional limitations from MG despite having some weakness on examination of some muscles) or better, as determined by the patient’s physician, with no more than grade 1 medication-related adverse effects according to the Common Terminology Criteria for Adverse Events [5, 12]. The patient-reported Myasthenia Gravis–Activities of Daily Living (MG–ADL) scale is commonly used in clinical practice to monitor the effect of treatment for MG; expanding on this measure, Vissing and colleagues developed the concept of minimal symptom expression (MSE) as a treatment target, with one definition of MSE being an MG-ADL total score of 0 or 1 [13].

Eculizumab (Soliris^®^, Alexion, AstraZeneca Rare Disease, Boston, MA, USA) is a humanized monoclonal antibody that binds with high affinity to human terminal complement protein C5, inhibiting its cleavage into C5a and C5b [14–16]. This prevents formation of the complement membrane attack complexes that architecturally damage the postsynaptic membrane of the neuromuscular junction [14–16]. In the phase 3 REGAIN clinical trial (NCT01997229), patients with refractory acetylcholine receptor (AChR) antibody–positive (Ab+) gMG who were treated with eculizumab for 26 weeks experienced clinically meaningful improvements in activities of daily living, muscle strength, functional ability, and quality of life [17]. The sustained efficacy and long-term safety of eculizumab were subsequently demonstrated in the REGAIN open-label extension (OLE; NCT02301624) [18]. Both eculizumab and the longer-acting C5 inhibitor ravulizumab have been approved by the United States (US) Food and Drug Administration (FDA) for the treatment of adults with AChR Ab+ gMG in the US.

The aim of the present study (R**E**al-wor**L**d **E**culizumab Effecti**V**eness and Imp**A**ct of Discon**T**inuation on US Pati**E**nt Outcomes in Myasthenia Gravis [ELEVATE]) was to examine treatment patterns and effectiveness of eculizumab in clinical practice for US patients with MG. Here we report baseline demographic data and findings from the study relating to symptom improvement and reduction in use of corticosteroids and NSISTs.

## Methods

### Study design

ELEVATE was an observational, retrospective, multisite medical chart review study conducted in the US. Data were collected from physicians in the Cardinal Health Neurology Provider Extended Network and other providers who were known to prescribe eculizumab, comprising both community practices and academic centers. Physicians abstracted their patients’ electronic medical record data for the study.

The index date was defined as the date of initiation of eculizumab treatment for MG. The pre-index period included the 24 months preceding eculizumab initiation; the post-index period was from the initiation of eculizumab until 24 months of follow-up or discontinuation, whichever occurred first.

#### Patient inclusion criteria

Eligible patients were adults (age ≥ 18 years) with a diagnosis of MG at the index date, who initiated commercially available eculizumab between October 23, 2017 (FDA approval date for patients with AChR Ab+ gMG), and December 31, 2019. Patients were currently or previously managed within a participating provider’s practice at the time of data collection (i.e., provider could report on complete diagnosis/treatment details, including treatment prescribed by other physicians, and inpatient treatment). No exclusion criteria were specified; refractory status was not an inclusion/exclusion criterion.

### Clinical outcomes

MG-ADL total scores were collected at the last assessment before eculizumab initiation and at approximately 3, 6, 12, and 24 months after initiation. The MG-ADL total scores over time were also analyzed in the subsets of patients who reduced or discontinued their prednisone dosage. The proportion of patients who achieved MSE (MG-ADL total score of 0 or 1) was calculated based on available MG-ADL data.

Physician impression of clinical change at the most recent assessment before eculizumab initiation, at initiation, and at approximately 3, 6, 12, and 24 months after initiation was assessed via a question in the data collection form: “Please indicate your (physician) impression of how your patient’s MG has changed over time with Soliris (eculizumab),” to which physicians could respond “worsened,” “unchanged,” or “improved.” For patients whose physicians reported an improvement in MG symptoms, achievement of MMS (defined as above) or better was reported if applicable.

### Concomitant medication use

The proportions of patients receiving concomitant therapies at the time of eculizumab initiation, and who had reduced or discontinued their prednisone dosage during eculizumab treatment, were determined. Median times to prednisone or NSIST dosage reduction or discontinuation during eculizumab treatment were calculated.

### Statistical analysis

Outcomes were summarized for the period before eculizumab initiation (up to 2 years) and the period during eculizumab treatment (up to 2 years). Univariate analyses compared mean clinical outcome scores before and at different timepoints after eculizumab initiation using paired *t* tests and chi-squared tests (Fisher’s exact test was applied where the sample size was fewer than five patients). Bonferroni corrections were not made for multiple comparisons. Median time to prednisone or NSIST dosage reduction or discontinuation was calculated using Kaplan–Meier methodology. A two-sided α value of 0.05 was used to determine statistical significance.

## Results

### Provider/practice characteristics

Fourteen experienced neurologists or neuromuscular specialists from 14 different sites, with a mean (standard deviation [SD]) time in practice of 18.5 (10.0) years, provided patient-level data for the study. Half of the providers were from academic centers (Table 1).

**Table 1:** Provider/practice characteristics

Characteristic	All providers (*n* = 14)
Duration in medical practice, years, mean (SD)	18.5 (10.0)
Primary practice setting, *n* (%)	
Academic center	7 (50.0)
Large practice (>10 physicians)	4 (28.6)
Medium practice (6–10 physicians)	2 (14.3)
Small practice (2–5 physicians)	0
Solo practitioners	1 (7.1)
US geographic region,* n* (%)	
Northeast^a^	1 (7.1)
South^b^	6 (42.9)
Midwest^c^	3 (21.4)
West^d^	4 (28.6)
Specialty, *n* (%)^e^	
Neurology	9 (64.3)
Neuromuscular neurology	9 (64.3)

*SD* standard deviation

^a^Northeast includes Connecticut, Delaware, Massachusetts, Maine, Maryland, New Hampshire, New Jersey, New York, Pennsylvania, Rhode Island, and Vermont

^b^South includes Alabama, Arkansas, District of Columbia, Florida, Georgia, Kentucky, Louisiana, Mississippi, North Carolina, Oklahoma, South Carolina, Tennessee, Texas, Virginia, and West Virginia

^c^Midwest includes Iowa, Illinois, Indiana, Kansas, Michigan, Minnesota, Missouri, North Dakota, Nebraska, Ohio, South Dakota, and Wisconsin

^d^West includes Alaska, Arizona, California, Colorado, Hawaii, Idaho, Montana, New Mexico, Nevada, Oregon, Utah, Washington, and Wyoming

^e^Categories not mutually exclusive

### Study population

In total, 119 patients with a diagnosis of MG were included in the ELEVATE study (Table 2); 60% of the patients were women and 16% were Black/African American. The mean (SD) age at diagnosis was 50.5 (20.7) years, with a bimodal distribution by sex: 43.4 (19.1) years and 61.0 (18.5) years for women and men, respectively. The mean (SD) age of patients at eculizumab initiation was 57.7 (17.7) years (Table 3). In general, men received eculizumab earlier after diagnosis than did women (mean time after diagnosis: 6.6 vs. 8.8 years, respectively).

**Table 2:** Patient demographics

Characteristic	Patients (*n* = 119)
Sex at birth, *n* (%)	
Male	48 (40.3)
Female	71 (59.7)
Age at MG diagnosis (years)	
Mean (SD)	50.5 (20.7)
Median (IQR)	53 (36–69)
Race,* n* (%)	
White	96 (80.7)
Black/African American	19 (16.0)
Asian	2 (1.7)
American Indian/Alaskan Native	1 (0.8)
Other	1 (0.8)
Insurance status at eculizumab initiation, *n* (%)^a^	
Medicare	62 (52.1)
Medicaid	10 (8.4)
Commercial	53 (44.5)
Military	2 (1.7)
Unknown	1 (0.8)

*IQR* interquartile range, *MG* myasthenia gravis, *SD* standard deviation

^a^Categories not mutually exclusive

**Table 3:** Patient clinical characteristics

Characteristic	Patients (*n* = 119)
Age at initiation of eculizumab therapy (years)	
Mean (SD)	57.7 (17.7)
Median (IQR)	59 (45–72)
Time from diagnosis to eculizumab initiation (years), mean (SD)	7.7 (9.8)
MG-ADL score before eculizumab initiation^a^	
Mean (SD)	8.0 (3.8)
Median (IQR)	8.0 (5.0–10.0)
MGFA classification at eculizumab initiation, *n* (%)	
Class I	12 (10.1)
Class II	30 (25.2)
Class III	36 (30.3)
Class IV	6 (5.0)
Class V	0 (0.0)
Unknown	35 (29.4)
AChR antibody status tested before eculizumab initiation, *n* (%)	118 (99.2)
Seropositive	114 (96.6)
Seronegative	3 (2.5)
Unknown	1 (0.8)
Meningococcal vaccination ≥14 days before eculizumab initiation, *n* (%)	
Yes	109 (91.6)
No^b^	10 (8.4)
Treatment for MG before eculizumab initiation^c^, *n* (%)	
Prednisone	73 (61.3)
NSIST	65 (54.6)
Rituximab	4 (3.4)
Pyridostigmine	95 (79.8)
Chronic IVIg	52 (43.7)
Chronic PLEX	12 (10.1)
None of the above	5 (4.2)

*AChR* acetylcholine receptor, *IQR* interquartile range, *IVIg* intravenous immunoglobulin, *MG* myasthenia gravis, *MG-ADL* Myasthenia Gravis-Activities of Daily Living, *MGFA* Myasthenia Gravis Foundation of America, *NSIST* nonsteroidal immunosuppressant therapy, *PLEX* plasma exchange, *SD* standard deviation

^a^Most recent score before eculizumab initiation; *n* = 92

^b^Of the 10 patients reported as not receiving meningococcal vaccination, two received prophylactic antibiotics in order to start eculizumab immediately and discontinued the antibiotics at least 14 days after vaccination, five patients were reported to have not received prophylactic antibiotics, and the prophylactic antibiotic status was unknown for three patients

^c^In the 24 months before eculizumab initiation

In this clinical practice setting, despite the Myasthenia Gravis Foundation of America (MGFA) classification at initiation of eculizumab therapy being recorded as Class I disease in 10% of patients (Table 3), 93% were recorded as having generalized disease involvement (i.e., gMG) at eculizumab initiation. Seven patients (6%) were recorded as having ocular MG; however, although three of these patients were recorded as having MGFA Class I disease, one as having Class II disease, and one as having Class IVa disease, two did not have a reported classification. MGFA classification data were missing for almost one-third (29%) of the study population.

Testing for AChR antibodies was performed in 118 of 119 patients (99%); a positive AChR antibody test result was reported in 97% of patients (Table 3). A history of thymoma was reported in 16 patients (13%), of whom 14 (12%) had had a thymectomy. Five patients (4%) were pregnant at the time of eculizumab initiation. As per the eculizumab US prescribing information (USPI) [19], 92% of patients received meningococcal vaccination ≥14 days before initiating eculizumab (Table 3). At the time of eculizumab initiation, the majority of patients (52%) had Medicare health insurance coverage and 45% had commercial insurance.

The majority of patients (83%) had comorbidities at eculizumab initiation, the most common of which were hypertension (35%), diabetes (24%) and cardiovascular disease (21%) (Online resource 1). By 24 months after eculizumab initiation, seven patients (6%) had died and three (3%) had been lost to follow-up. No meningococcal infections were reported in the study population.

Mean (SD) MG-ADL total score was 8.0 (3.8) before eculizumab initiation (median 8.0, interquartile range [IQR] 5.0; data available for 92 patients [77%]).

### Eculizumab use

**Fig. 1:**
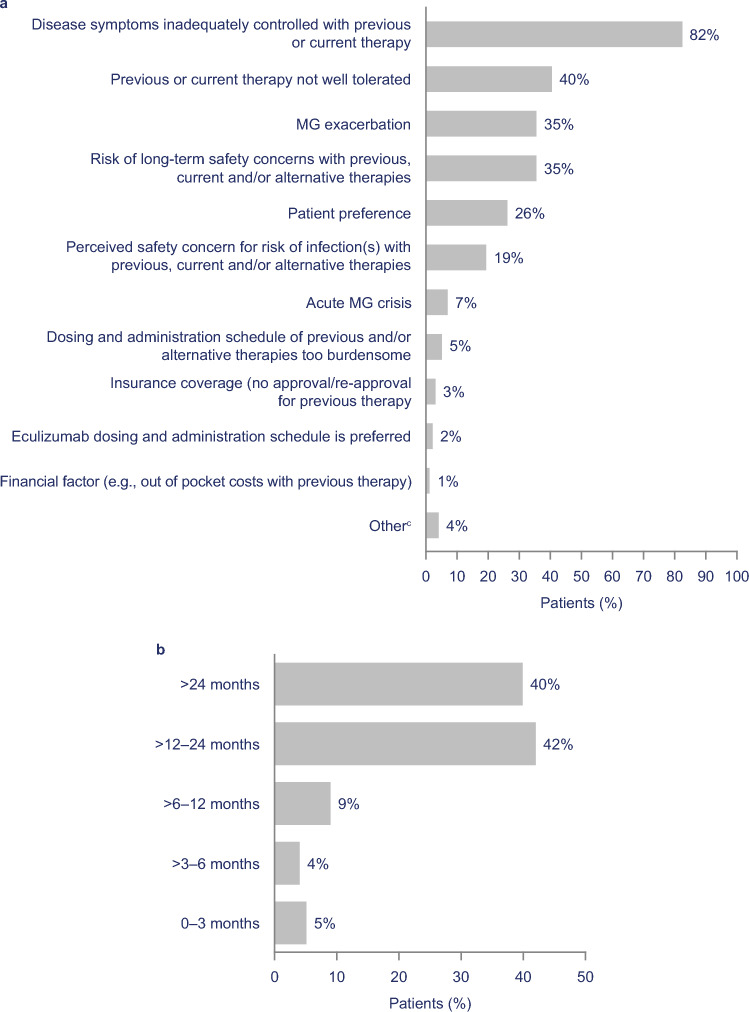
Eculizumab use: **a** reasons for initiating eculizumab^a^ (*n* = 119); **b** duration of eculizumab treatment at data cut-off^b^ (*n* = 119). ^a^Categories not mutually exclusive. ^b^Of patients who were alive at the time of data cut-off (*n* = 109), 88% were receiving eculizumab. ^c^Providers were allowed to specify other reasons, which included patients with an impending crisis. *MG* myasthenia gravis

The most frequent reason given by physicians for initiating eculizumab was inadequate control of symptoms with previous or current therapy (82%) (Fig. 1a). During the study period, most patients (*n* = 97; 82%) were treated with eculizumab for at least 1 year (Fig. 1b). At last follow-up after eculizumab initiation, 88% (96/109 patients with data) were still receiving eculizumab. Of the 119 patients treated with eculizumab, 108 (91%) received it per the USPI with no dosage modifications; six (5%) had their dosing interrupted temporarily (i.e., missed ≤ 2 doses), four (3%) received fewer infusions (longer interval between doses), and one (1%) received more infusions (shorter interval between doses). Twenty patients (17%) discontinued eculizumab therapy (i.e., missed ≥3 consecutive doses) (Online resource 2).

### Eculizumab effectiveness

#### MG symptoms

**Fig. 2:**
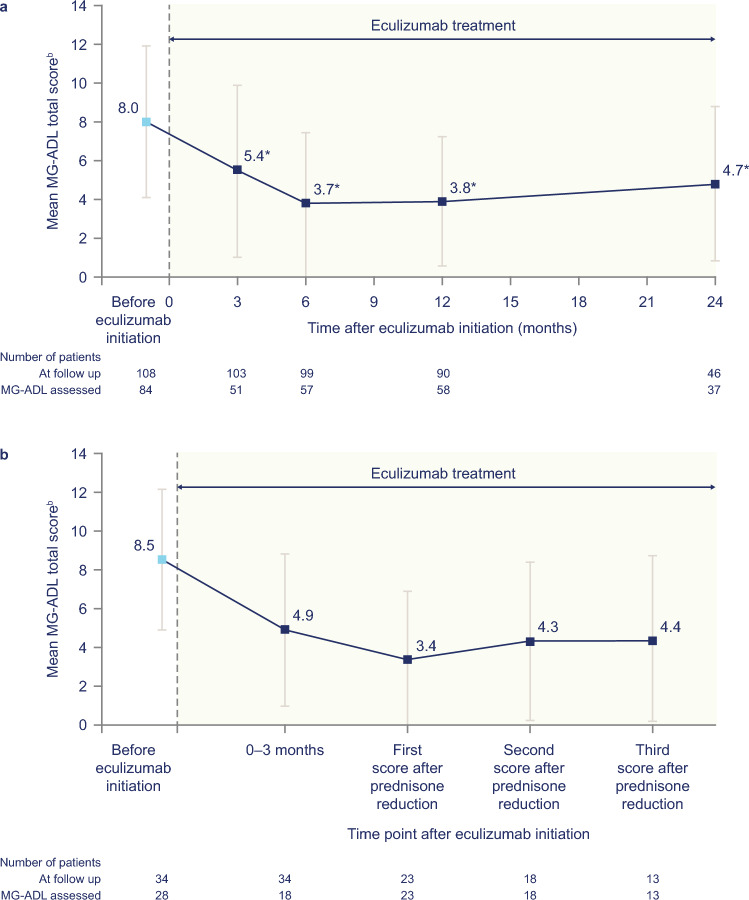
Mean (±SD) MG-ADL total scores over time in **a** patients who received eculizumab per the prescribing information in the United States, and **b** the subset of patients with prednisone dosage reduction and ≥12 months of follow-up^a^. ^a^Statistical analysis not conducted. ^b^Lower scores indicate less severe symptoms. * *p* < 0.001 versus mean MG-ADL scores before eculizumab initiation (paired *t* test). *MG-ADL* Myasthenia Gravis–Activities of Daily Living, *SD* standard deviation

In patients who received eculizumab per the USPI and had MG-ADL assessments (*n* = 84), the mean MG-ADL total score at 3 months (5.4) was statistically significantly improved (*p* < 0.001) compared with the mean total score at eculizumab initiation (8.0), and this was sustained through 24 months of eculizumab treatment (4.7 at 24 months) (Fig. 2a). A similar pattern of improvement in MG-ADL scores was seen in a subset of patients whose prednisone dosage was reduced after eculizumab initiation (Fig. 2b) and in those who discontinued prednisone (*n* = 9; data not shown).

**Fig. 3:**
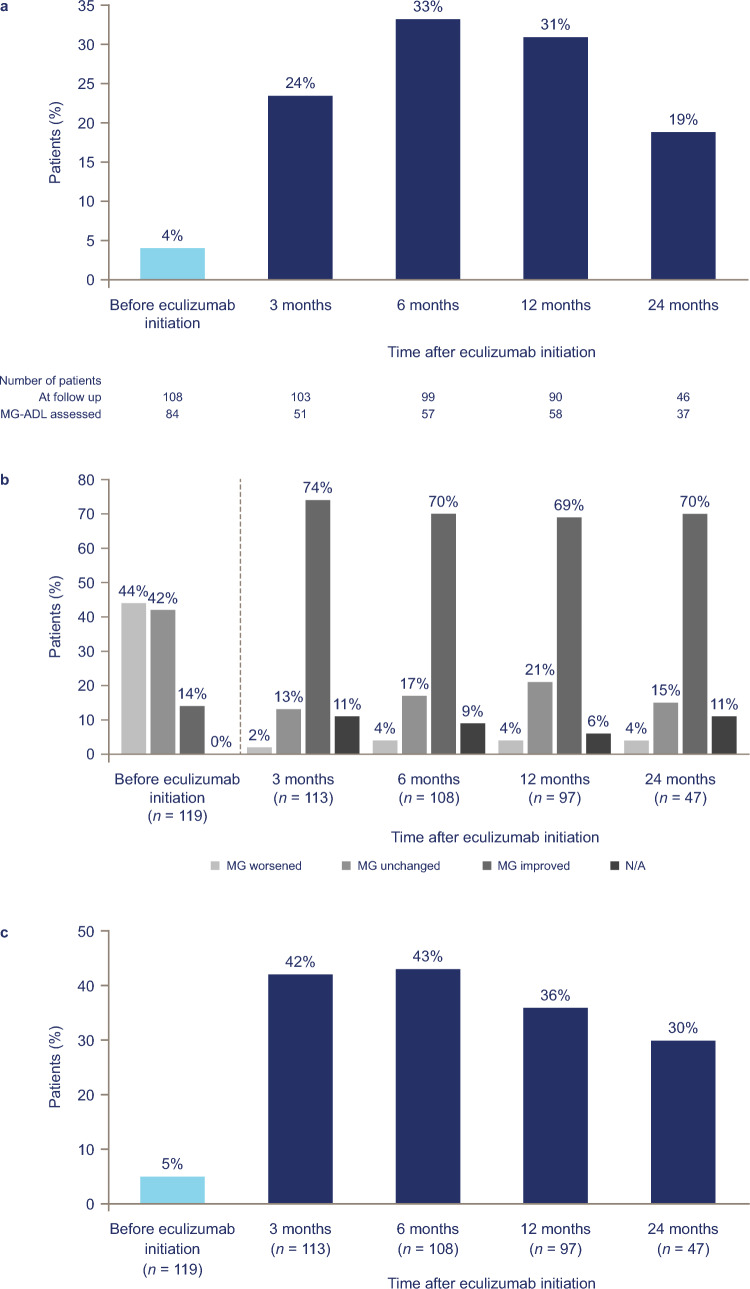
**a** Minimal symptom expression^a^ over time in patients in the study population who received eculizumab per the prescribing information; **b** physicians’ impression of change in patients’ disease; and **c** minimal manifestation status or better^b^ before and during eculizumab treatment. ^a^MG-ADL score of 0 or 1. ^b^Minimal manifestation status is based on the percentage of the total patient population with available data; minimal manifestation status was defined as having MG improvement and no symptoms or functional limitations from MG, but some weakness on examination of some muscles. *MG-ADL* Myasthenia Gravis–Activities of Daily Living, *N/A* not applicable (includes patients who were no longer receiving eculizumab or did not have data)

Only 4% (3/84) of patients had achieved MSE in the 2-year period before eculizumab initiation. After eculizumab initiation, the proportion of patients receiving the approved USPI dosage who achieved MSE increased to 24% (12/51) at 3 months, 33% (19/57) at 6 months, and 31% (18/58) at 12 months; it was 19% (7/37) at 24 months (Fig. 3a).

#### Physician impression of clinical change

Physicians reported that MG improved after initiating eculizumab in most patients. By 3 months after initiating eculizumab, MG had improved in 74% of patients compared with 14% in the period up to 2 years before eculizumab initiation, and the proportion was maintained at 70% through 24 months of eculizumab treatment (Fig. 3b).

#### Minimal manifestation status

MMS or better was achieved in a numerically higher proportion of patients receiving eculizumab at 6 months (46/108, 43%), 12 months (35/97, 36%), and 24 months (14/47, 30%) after initiation compared with before eculizumab initiation (6/119, 5%) (Fig. 3c).

### Concomitant medication use

**Fig. 4:**
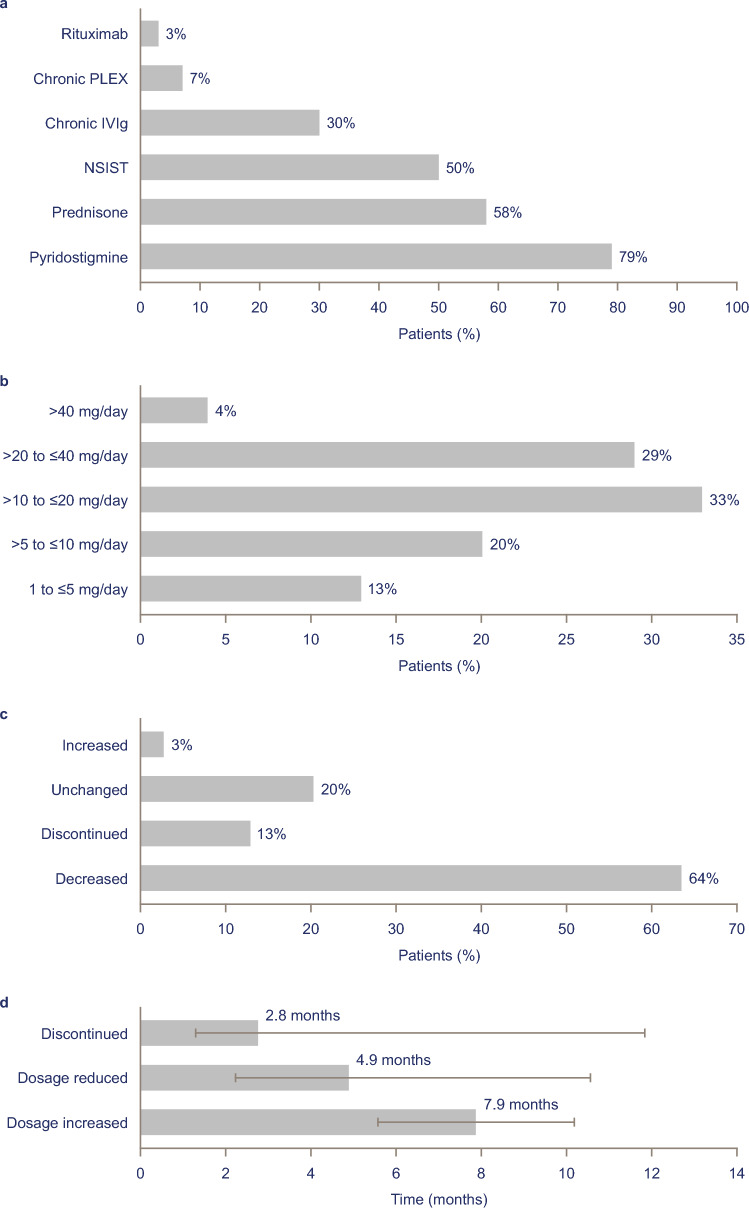
Concomitant therapies: **a** use of concomitant therapies^a^ at eculizumab initiation (*n* = 119); **b** prednisone dosage at eculizumab initiation (*n* = 69); **c** proportion of patients with changes in prednisone dosage during eculizumab treatment (*n* = 69); **d** median (interquartile range) time to change in prednisone dosage during eculizumab treatment^b^ (*n* = 69). Cited values may not sum to 100% because of rounding. ^a^Patients may have been receiving more than one concomitant therapy. ^b^Time to discontinuation may be impacted by prednisone dosage—higher dosages may have required more gradual tapering. *IVIg* intravenous immunoglobulin, *PLEX* plasma exchange, *NSIST* nonsteroidal immunosuppressant therapy

At the time of eculizumab initiation, 69 patients (58%) were receiving prednisone and 60 (50%) were receiving NSISTs (Fig. 4a). Overall, 67% of those receiving prednisone were receiving >10 mg/day; 33% were receiving >20 mg/day (Fig. 4b). Of patients receiving prednisone at the time of eculizumab initiation, 64% (44/69) were able to reduce their prednisone dosage during eculizumab treatment (Fig. 4c); of those, 52% (23/44) were able to decrease their dosage to ≤10 mg/day. In addition, 13% of patients (9/69) discontinued prednisone. Dosage data were missing for 17/44 patients (39%) who were known to have reduced their prednisone use. Median (IQR) time to prednisone dosage reduction and discontinuation was 4.9 (8.4) months and 2.8 (10.7) months, respectively (Fig. 4d). In total, 5% of patients (3/60) receiving NSISTs at the time of eculizumab initiation had their NSIST dosage reduced, and 32% of patients (19/60) discontinued NSIST during eculizumab treatment. Median (IQR) time to NSIST dosage reduction was 11.3 (11.2) months; median (IQR) time to NSIST discontinuation was 4.8 (10.5) months.

## Discussion

This retrospective chart review demonstrated the benefits associated with eculizumab treatment in routine clinical practice in patients with MG in the US, with regard to improvement in disease symptoms and reduction in the use of concomitant prednisone and NSISTs.

Of the quantitative clinical outcome assessments examined, sufficient data for analysis were available for MG-ADL total scores only. In the study population, a statistically significant and clinically meaningful (i.e., ≥2 points) [20] reduction in MG-ADL total score was observed at 3 months after eculizumab initiation and sustained through 24 months. These data are consistent with those reported in other studies of eculizumab treatment in patients with gMG in clinical practice. Several retrospective chart reviews and case-series studies in patients with AChR Ab+ gMG (mostly defined as being treatment refractory) in the US, Canada, and Japan have reported rapid and sustained clinically meaningful responses to eculizumab [8, 21–25]. A larger, post-marketing surveillance study in Japan reported sustained improvements in MG-ADL scores through 1 year of eculizumab treatment, including in patients with a history of thymoma [26, 27]. An analysis of clinical practice data from a US gMG registry found that MG-ADL scores improved by at least 3 points in 64% of patients treated with eculizumab for a mean of approximately 1.8 years [28]. In addition, a recent case-series review of seven patients in the US with refractory gMG reported that patients who had stopped or changed their work/school hours after MG diagnosis were able to return to work/school after eculizumab initiation [22]. Overall, these real-world effectiveness results are comparable with results seen in REGAIN [17] and its OLE [18], which demonstrated sustained efficacy of eculizumab through 3 years.

In the ELEVATE study, the proportion of patients with MSE increased after eculizumab initiation. This is in accordance with the REGAIN OLE outcomes, in which the proportion of patients with MSE in the group that switched from placebo to open-label eculizumab increased within 4 weeks after switching, to reach a level similar to that in the group who received eculizumab throughout the study [18].

Physicians’ impressions of change in disease status following eculizumab initiation indicated that improvements had occurred in approximately three-quarters of patients when assessed at 3 months and were sustained for the duration of treatment through 24 months. Physician impression of change has been shown to be significantly correlated with the change in patient-reported MG-ADL score, which reflects patients’ own assessments of the impact of their symptoms on their daily activities in the past 7 days [29]. Thus, the observed improvements in disease status after eculizumab initiation are consistent with the statistically significant and clinically meaningful reductions in mean MG-ADL total scores.

A task force of the Medical Scientific Advisory Board of the MGFA developed the concept of post-intervention status (MGFA-PIS) in 2000 to evaluate MG outcomes after initiation of treatment in routine clinical practice and clinical trials [12]. The MGFA-PIS assesses changes in clinical status and category of remission achieved (complete stable, pharmacologic, or with minimal manifestations of disease) based on the patient’s treatment and the physician’s assessment of the patient’s functional weakness over time [12]. In the current study, the proportion of patients who achieved MMS or better increased after eculizumab initiation (from 5% before initiation to 43% at 6 months after initiation). This result is comparable with an analysis of MGFA-PIS in patients treated with eculizumab during REGAIN, which showed that 37% of patients had achieved MMS or better at Week 26 [30]. Rapid improvements in MGFA-PIS were also noted in a small US chart review study in patients with AChR Ab+ gMG who were treated with eculizumab [25]. A decrease in the proportion of patients who achieved MMS or better was observed at 12 and 24 months compared with 6 months after eculizumab initiation in the current study; the reason for this is unclear but may reflect the smaller number of patients with available data at those time points, possibly partly due to discontinuation by patients whose symptoms improved.

Reduction in the burden of concomitant medication, particularly corticosteroids, is an important treatment goal based on minimizing the risks of adverse effects of these agents [6, 9], which include diabetes, hypertension, and osteoporosis [7, 8]. In the current study, almost two-thirds of patients treated with prednisone reduced their prednisone dosage while being treated with eculizumab; at least 32 patients (46%) were able to decrease their dosage to ≤10 mg/day (including nine patients who discontinued prednisone). This finding is consistent with previous and ongoing clinical practice studies that have reported a reduction in daily oral corticosteroid dose after eculizumab initiation [8, 24–26, 31]. It is also comparable with results from the REGAIN OLE, which reported that 47.9% of patients who used prednisone during the OLE decreased and/or stopped their prednisone [32]. An additional key finding in the current study was that the improvement in MG-ADL total scores over time was maintained in the subset of patients whose prednisone dosage had been reduced. The data indicate that, in the current study, the median time to corticosteroid discontinuation was shorter than that for dosage reduction. The reason for this is not clear but might be due to differences in dosages between these two subgroups: a higher proportion (78%) of patients who discontinued steroids than those who reduced their dosage (54%) were receiving <20 mg/day while being treated with eculizumab. The dosage may have impacted the time to discontinuation: those patients receiving higher dosages may have been gradually tapered down toward discontinuation.

Use of NSISTs is also associated with adverse effects, including increased susceptibility to serious infections, a wide range of deleterious systemic effects (such as nephrotoxicity, neuropathy, and hypomagnesemia), and increased long-term risk of malignancy [33, 34]. In the current study, approximately one-third of patients discontinued NSISTs during eculizumab treatment. Overall, the data presented here from US clinical practice supplement evidence from the REGAIN OLE that eculizumab reduces the treatment burden associated with corticosteroids and NSISTs and has steroid-sparing benefits [32].

The ELEVATE patient population was clinically and demographically more diverse than that of the phase 3 REGAIN study [17]. Whereas no Black/African American patients were randomized to the eculizumab arm in REGAIN, 16% of patients in ELEVATE were Black/African American, which is more reflective of the current US population [35]. Additionally, 10% of patients were reported to have MGFA Class I disease, which was an exclusion criterion in the REGAIN study, and 13% had a history of thymoma (including patients who had undergone thymectomy), whereas patients with thymoma, thymic neoplasms, or thymectomy were excluded from REGAIN. In ELEVATE, 3% of patients were AChR Ab-negative, in contrast to the REGAIN population in which patients were exclusively AChR Ab+. Also of note, patients evaluated in the ELEVATE study may have had MG defined by their physicians as “refractory” or “nonrefractory”; refractory status was not an inclusion/exclusion criterion for the study as it is not a condition of the MG indication in the USPI [19]. This is in contrast to the REGAIN study population, which enrolled patients with gMG defined by the study protocol as being refractory to treatment [17]. The mean ages at diagnosis and at eculizumab initiation were somewhat higher in patients in the ELEVATE study than in REGAIN. The disease and demographic characteristics of patients included in this study may not be consistent with those of the study population in REGAIN so comparison of outcomes between the studies should be interpreted with caution; however, the findings from ELEVATE provide evidence that eculizumab is effective in a broad population with MG.

Overall, the findings presented here provide evidence from clinical practice that eculizumab is effective across a spectrum of patients who have MG with varying characteristics and medical histories. A phase 3 clinical trial of ravulizumab (CHAMPION MG, NCT03920293) confirmed the efficacy and good safety profile of complement C5 inhibitors [36, 37], and a recently reported postmarketing surveillance study of eculizumab across three indications (paroxysmal nocturnal hemoglobinuria, atypical hemolytic uremic syndrome, and AChR Ab+ gMG) in over 1000 patients in Japan over a period of more than 10 years has confirmed the safety profile of eculizumab in clinical practice to be consistent with that in clinical trials [38]. An ongoing registry study (NCT04202341) will provide further information on the use of both eculizumab and ravulizumab in clinical practice [28, 31] for the treatment of AChR Ab+ gMG.

The results reported here are based on medical record data from a large sample of patients treated with eculizumab, drawn from a wide range of practice settings and geographic locations in the US. Longitudinal data on clinical outcomes are reported over multiple timepoints through 24 months. Additionally, the pre- versus post-eculizumab study design reduced the effects of patient heterogeneity because the patients served as their own controls.

This study has several limitations: patients had variable follow-up periods at the time of data cut-off; MGFA classification data were missing in almost one-third of patients; and the endpoint of MSE has not yet been formally validated as an outcome measure in patients with MG. In particular, MG-ADL scores were not available for all patients at all timepoints: some patients had shorter follow-up times.  Additionally, although 82% of patients were still receiving eculizumab after 1 year (Fig. 1b), MG-ADL scores were not recorded in a considerable number of patients; the reasons for lack of assessment are not known. Limited data were available for other clinical outcomes, including Quantitative Myasthenia Gravis score (5% of patients), Myasthenia Gravis Composite score (13%), and the revised 15-item Myasthenia Gravis Quality of Life Questionnaire (29%); thus, these assessments were not analyzed. This study confirms that such instruments are less frequently applied than the MG-ADL scale in clinical practice [39, 40]; however, their application would provide a more comprehensive assessment of treatment effectiveness. The study did not include a comparator group (for example, patients receiving standard care); the pre/post study design permitted outcomes to be compared before and during eculizumab treatment, but did not control for longitudinal variation in other factors. Other limitations include those inherent in retrospective medical chart reviews: data were abstracted from electronic medical records, and as such were limited to what was recorded in consultations and were subject to variation between healthcare providers and timepoints. Although causal relationships between treatment and outcomes cannot be established from this study, the results support those of the rigorous clinical trial and of other real-world studies of eculizumab.

## Conclusions

In this study of US clinical practice, patients with MG treated with eculizumab experienced clinically meaningful and sustained improvements (up to 2 years) in their MG-ADL total scores. Physicians were able to reduce the prednisone dosage in two-thirds of patients, and this subset of patients experienced improvements in MG-ADL scores that were maintained and similar to those seen in the overall population, suggesting the potential for eculizumab treatment to have steroid-sparing effects. Almost one-third of patients receiving NSIST at the time of eculizumab initiation were able to discontinue NSIST during eculizumab treatment. Overall, despite differences in study design, these data from clinical practice are in accordance with findings from the phase 3 REGAIN study and its OLE, and with other real-world studies, in demonstrating the sustained benefit of eculizumab in the treatment of patients with MG.

## Supplementary Information

Below is the link to the electronic supplementary material.

Supplementary file1 (DOCX 63 KB)

## Data Availability

Alexion, AstraZeneca Rare Disease will consider requests for disclosure of clinical study participant-level data provided that participant privacy is assured through methods like data de-identification, pseudonymization, or anonymization (as required by applicable law), and if such disclosure was included in the relevant study informed consent form or similar documentation. Qualified academic investigators may request participant-level clinical data and supporting documents (statistical analysis plan and protocol) pertaining to Alexion-sponsored studies. Further details regarding data availability and instructions for requesting information are available in the Alexion Clinical Trials Disclosure and Transparency Policy at https://www.alexionclinicaltrialtransparency.com. Link to Data Request Form: https://contactazmedical.astrazeneca.com/content/astrazeneca-champion/us/en/amp-form.html.
