# Supplementary material for: United States clinical practice experience with eculizumab in myasthenia gravis: symptoms, function, and immunosuppressant therapy use
Source: J Neurol. 2024 Jul 25;271(9):6114–26. doi: 10.1007/s00415-024-12569-w (PMC11377470; doi:10.1007/s00415-024-12569-w)
Supplement: Supplementary file 1 — Supplementary file1 (DOCX 63 KB) [file 415_2024_12569_MOESM1_ESM.docx]

**Supplementary material**

**United States clinical practice experience with eculizumab in myasthenia gravis: symptoms, function, and immunosuppressant therapy use**

Journal of Neurology

Ali A. Habib^1^, Andrew J. Klink^2^, Srikanth Muppidi^3^, Anju Parthan^4,a^, S. Chloe Sader^4^, Alexandrina Balanean^2^, Ajeet Gajra^2,b^, Richard J. Nowak^5^, James F. Howard Jr^6^, and the ELEVATE Study Group

*^1^University of California, Irvine, CA, USA*

*^2^Cardinal Health, Dublin, OH, USA*

*^3^Stanford Neuroscience Health Center, Palo Alto, CA, USA*

*^4^Alexion, AstraZeneca Rare Disease, Boston, MA, USA*

*^5^Yale University School of Medicine, New Haven, CT, USA*

*^6^The University of North Carolina, Chapel Hill, NC, USA*

^a^Current affiliation: FibroGen Inc., San Francisco, CA, USA

^b^Current affiliation: Hematology-Oncology Associates of CNY, E. Syracuse, NY, USA

**Correspondence**
Dr. JF Howard Jr, Department of Neurology, The University of North Carolina, Chapel Hill, NC, USA. Email: [howardj@neurology.unc.edu](mailto:howardj@neurology.unc.edu)

**Table S1** Comorbidity at eculizumab initiation (*N* = 119)

| **Condition^a^** | **No. (%) of patients** |
| --- | --- |
| Hypertension | 42 (35.3) |
| Diabetes | 29 (24.4) |
| Cardiovascular disease | 25 (21.0) |
| Osteoarthritis | 21 (17.6) |
| Depression | 21 (17.6) |
| Thyroid disorder | 18 (15.1) |
| Respiratory disease | 10 (8.4) |
| Malignancies | 7 (5.8) |
| Osteoporosis | 6 (5.0) |
| Renal disease | 6 (5.0) |
| Anemia | 5 (4.2) |
| Gastric disease | 5 (4.2) |
| Osteopenia | 4 (3.4) |
| Vitamin deficiency | 4 (3.4) |
| Obesity | 4 (3.4) |
| Inflammatory bowel disease | 3 (2.5) |
| Anxiety | 3 (2.5) |
| Autoimmune thyroiditis | 2 (1.7) |
| Multiple sclerosis | 2 (1.7) |
| Psoriasis | 2 (1.7) |
| Systemic lupus erythematosus | 2 (1.7) |
| Thyroid disease | 2 (1.7) |
| Diabetic neuropathy | 1 (0.8) |
| Glomerulonephritis | 1 (0.8) |
| Graves disease | 1 (0.8) |
| Guillain-Barré syndrome | 1 (0.8) |
| Idiopathic thrombocytopenia | 1 (0.8) |
| Neuromyelitis optica spectrum disorder | 1 (0.8) |
| Primary biliary cirrhosis | 1 (0.8) |
| Rheumatoid arthritis | 1 (0.8) |
| Sexually transmitted disease | 1 (0.8) |
| Other | 12 (10.1) |
| None of the above | 20 (16.8) |

^a^Physicians were asked specifically to indicate whether patients had any of the conditions listed, or any of following (all *n* = 0): alopecia areata, autoimmune hepatitis, dermatomyositis, granulomatosis with polyangiitis (Wegener’s), pemphigus/pemphigoid, polyarteritis nodosa, polymyositis, scleroderma/systemic sclerosis, Sjögren syndrome, vitiligo, fibromyalgia, or ‘other’.

**Table S2** Reasons for eculizumab discontinuation (*n* = 20)

| **Reason^a^** | **No. patients** |
| --- | --- |
| Therapy not well tolerated | 6 |
| Patient has improved clinically | 4 |
| Disease symptoms inadequately controlled | 3 |
| Patient preference | 3 |
| Financial factors (e.g., out of pocket costs) | 3 |
| MG exacerbation | 1 |
| Acute MG crisis | 1 |
| Insurance coverage (no re-approval) | 1 |
| Other | 2 |

^a^More than one reason may be recorded for each person who discontinued

*MG* myasthenia gravis
